# Clinical Features and Risk Factors Analysis for Hemorrhage in Adults on ECMO

**DOI:** 10.3389/fmed.2021.731106

**Published:** 2021-12-14

**Authors:** Wenwen Hu, Jing Zhang, Meifang Wang, Wei Chen, Lin Chai, Elaine Lai-Han Leung, Yijun Tang

**Affiliations:** ^1^Department of Neurological Intensive Care Unit, Taihe Hospital, Shiyan, China; ^2^Department of Respiratory and Critical Care Medicine, Taihe Hospital, Hubei University of Medicine, Shiyan, China; ^3^Department of Critical Care Medicine, Taihe Hospital, Hubei University of Medicine, Shiyan, China; ^4^Department of Emergency Medicine, Taihe Hospital, Shiyan, China; ^5^State Key Laboratory of Quality Research in Chinese Medicine, Macau Institute for Applied Research in Medicine and Health, Macau University of Science and Technology, Macau, China

**Keywords:** extracorporeal membrane oxygenation, hemorrhage, bleeding, complication, blood transfusion

## Abstract

**Background:** The use of extracorporeal membrane oxygenation (ECMO) to support critically ill patients with cardiorespiratory dysfunction has increased over the last decades. However, hemorrhagic complications occur frequently during ECMO support, and this has a significant impact on morbidity and mortality. Thus, this study aimed to identify the risk factors for hemorrhage in patients receiving ECMO.

**Methods:** Our retrospective study included 60 patients, who were admitted to the Taihe Hospital in Shiyan City, Hubei Province, China from February 2017 to October 2020. About 18 patients developed hemorrhagic complications, and 42 patients did not demonstrate such complications. Data regarding patient demography, laboratory tests, and clinical manifestations prior to ECMO were collected to analyze their clinical features. Univariable and multivariable logistic analyses were used to explore the risk factors for hemorrhage in adults on ECMO. The receiver operating characteristic (ROC) curve was used to evaluate the predictive value of the binary logistic model. The amount of blood transfusions was compared between the two groups, and the activated partial thromboplastin time (APTT), platelet count, and hemoglobin level before the initiation of ECMO.

**Results:** Logistic analysis showed that a longer duration of ECMO support, higher APTT, and lower platelet count prior to ECMO were independent risk factors for hemorrhage in adults on ECMO. In addition, we found that the cannula site was the most common bleeding site. Most bleeding events occurred within the first 3 days of ECMO therapy. After the ECMO initiation, APTT was prolonged while the platelet count and hemoglobin levels were decreased. The amount of blood transfusion was significantly higher in the hemorrhage group than in the non-hemorrhage group.

**Conclusions:** Clinicians should evaluate the risk of hemorrhage based on the coagulation function of patients, underlying disease, and the duration of ECMO support. In the first 3 days during ECMO support, special attention should be given to the cannula site, mucosal, and dermal regions, and digestive tract to detect any signs of hemorrhage. Moreover, increasing the platelet count transfusion threshold and accurately determining the amount of blood transfusion required may prevent bleeding events.

## Introduction

Extracorporeal membrane oxygenation (ECMO) is an advanced life support technique used to provide respiratory and cardiac support. ECMO has been used to save the lives of many critically ill patients with A/H1N1 influenza and coronavirus disease 2019 (COVID-19) as an important rescue therapy (1, 2). ECMO is invasive and is historically associated with notable complications. Hemorrhage is an important and very common complication in patients receiving ECMO support and causes a significant increase in mortality risk (3).

The activation of procoagulant and anticoagulant factors when a cannula makes contact with the endothelial surface of the blood vessels is associated with a risk of thrombosis (4). The Extracorporeal Life Support Organization (ELSO) anticoagulation guidelines recommend the use of antithrombotic therapy during ECMO (5). However, bleeding events can result from anticoagulation, and predicting the potential bleeding risk of patients is significant for determining the prognosis in adults on ECMO. Thus, this study aimed to identify the risk factors for hemorrhage in patients receiving ECMO.

## Materials and Methods

### Patients and Data Collection

Sixty patients admitted to Taihe Hospital in Shiyan City, Hubei Province, China from February 2017 to October 2020 were analyzed in this retrospective study. Data regarding demography, chronic medical histories, reason for ECMO therapy, Acute Physiology and Chronic Health Evaluation II (APACHE II) score, time from onset to ECMO initiation, duration of ECMO support, ECMO mode, organ function, laboratory values, prognosis, blood transfusions, hemorrhage sites, and time from ECMO initiation to hemorrhage were collected. We divided the 60 patients into two groups based on the presence or absence of hemorrhage. Those without hemorrhage were set as the control group (*n* = 42), whereas those with hemorrhage were set as the case group (*n* = 18). All patients received a therapeutic regimen that adhered to standardized management guidelines in accordance with the current ELSO recommendations or with the recommendations of similar medical societies. All bleeding events documented within the clinical data management system were retrospectively analyzed in the 60 patients. Hemorrhagic complications were subdivided into different categories according to the locations of the bleeding sites, including cannulation sites, surgical wounds, mucosal or dermal bleeding, gastrointestinal bleeding, respiratory tract bleeding, intracranial bleeding, intra-abdominal bleeding, or bleeding from other organs.

All patients were required to meet the following inclusive criteria: (1) patients treated with ECMO at our hospital (including those transferred to our hospital after ECMO was started in other hospitals); (2) age ≥ 18 years; (3) use of unfractionated heparin (UNFH) alone as the anticoagulant during ECMO; and (4) availability of complete clinical data.

Indications for ECMO were derived according to the 2017 ELSO General Guidelines for All Extracorporeal Life Support (ECLS) cases (3) as follows: (1) severe acute cardiopulmonary failure, where conventional treatment was ineffective and improvement or recovery was subsequently expected, or where the patient had undergone appropriate follow-up measures; (2) cardiogenic shock in acute myocardial infarction; (3) cardiogenic shock after cardiac surgery; (4) acute myocarditis; (5) transition of transplantation or ventricular assist; (6) acute respiratory distress syndrome; (7) lung transplantation; (8) severe pneumonia; (9) resuscitation of acute pulmonary embolism; and (10) unavailability of a heartbeat donor.

Bleeding was defined based on the 2014 ELSO Anticoagulation Guidelines 2014 (5). Major bleeding was defined as clinically overt bleeding associated with a decrease in hemoglobin of ≥2 g/dl within 24 h, >20 ml/kg within 24 h, or a transfusion of one or more 10 ml/kg units of packed red blood cells (PRBCs) over that same time period. Moreover, bleeding that was retroperitoneal, pulmonary, involved the central nervous system, or required surgical intervention was also considered major bleeding. Minor bleeding was considered as bleeding <20 ml/kg/day or requirement for one 10 ml/kg unit of PRBC or less.

The anticoagulant protocol was based on the 2014 ELSO Anticoagulation Guidelines (5). Patients usually received an initial UNFH bolus of 50–100 units/kg body weight at the time of cannulation for ECLS, and then UNFH was subsequently administered as a continuous infusion during the ECLS course. When the measured activated clotting time (ACT) dropped to ≤ 300 s, the UNFH infusion was typically initiated at a dose of 7.5–20 units/kg/h, with a lower dose range in adults. The bolus dose was adjusted based on clinical factors such as evidence of preexisting bleeding, and if the patient had recently undergone surgery or a cardiopulmonary bypass, the bolus dose was adjusted depending on whether the UNFH administered during the bypass had been reversed to any degree with protamine.

Blood transfusion indications referred to the 2017 ELSO General Guidelines for all ECLS cases (3). Cryoprecipitate was given if the fibrinogen level was <150 mg/dl. Frequent platelet transfusions of 10 ml/kg were administered to maintain a platelet count of >100 × 10^9^/L. The threshold for platelet transfusion was considered for the reduction in older patients with a lower risk of intracranial hemorrhage and was stable on ECLS. Thresholds for the transfusion of PRBC vary by institution and patient, but transfusions are generally administered as needed to replace any blood loss and to maintain a near-normal hematocrit (35–40%). The threshold for platelet transfusion in our hospital is a platelet count of <30 × 10^9^/L. The threshold for PRBC transfusion is a hemoglobin level of <100 g/L for patients with heart disease and 75 g/L for other patients. PRBC transfusion was considered if hemoglobin <90 g/L for patients with major bleeding.

### Statistical Analysis

Normally and non-normally distributed continuous variables are expressed as mean ± SD and median (interquartile range, IQR), respectively. We compared means for continuous variables using the independent *t*-test when the data were normally distributed; otherwise, we used the Mann–Whitney *U*-test. Categorical variables are expressed as numbers (%). We compared proportions for unordered categorical variables using the χ^2^-test or Fisher's exact test and compared proportions for ordered categorical variables using the Wilcoxon rank-sum test. Univariable and multivariable logistic regression models were used to explore the risk factors for hemorrhage in adults on ECMO. Considering the total number of patients (*n* = 60) in our study and to avoid multicollinearity in the model, six variables were chosen for multivariable analysis based on previous findings and clinical constraints. The receiver operating characteristic (ROC) curve was used to evaluate the predictive ability of the binary logistic model. We compared the blood transfusions between the two groups and analyzed the results of activated partial thromboplastin time (APTT), platelet count, and hemoglobin level during the treatment. For unadjusted comparisons, a two-sided α of <0.05 was considered statistically significant. Statistical analyses were conducted using the IBM SPSS version 4.0 (IBM Corp., Armonk, NY, USA).

## Results

### Demographics Characteristics

The demographic and clinical characteristics of the 60 patients who received ECMO support are shown in Table 1. We compared the age, sex, and medical history (including antiplatelet and anticoagulant history) of the hemorrhage and no-hemorrhage groups. There was no significant difference between the two groups. More than half of the 60 patients had comorbidities, including hypertension, diabetes, chronic obstructive pulmonary disease, preexisting cardiac disorder, chronic kidney diseases, and cancer. There were also no significant differences between the two groups regarding comorbidities.

**Table 1:** Demographics and clinical characteristics of patients.

**Variable**	**Hemorrhage Group (*n* = 18)**	**No-hemorrhage Group (*n* = 42)**	***P*** **-value**
Age, years	48.5 ± 11.7	48.0 ± 14.6	0.899
Gender			0.231
Male	12 (66.7%)	34 (81.0%)	
Female	6 (33.3%)	8 (19.0%)	
**Comorbidities**			
Hypertension	9 (50.0%)	13 (40.1%)	0.161
Diabetes	2 (11.1%)	2 (4.8%)	0.576
Preexisting Cardiac Disorder	8(44.4%)	12 (28.6%)	0.232
Chronic Obstructive Pulmonary Diseases	3 (16.7%)	1 (2.4%)	0.077
Chronic Kidney Diseases	2 (11.1%)	0 (0)	0.086
Cancer	1 (5.6%)	1 (2.4)	0.514
Anti-platelet or anticoagulant history	7 (38.9%)	15 (35.7%)	0.859
**Cause of ECMO Therapy**			
Cardiogenic Shock	7 (38.9%)	25 (59.5%)	0.142
Respiratory failure	4(22.2%)	2 (4.8%)	0.060
Infectious shock	3 (16.7%)	1(2.4%)	0.077
Traumatic shock	2 (11.1%)	5 (11.9%)	1.000
Electrical injury	0 (0)	2 (4.8%)	1.000
Intoxication	0 (0%)	1 (2.4%)	1.000
Aortic dissection	1 (5.6%)	3 (7.1%)	1.000
Others	1 (5.6%)	3 (7.1%)	1.000
APACHE II	26.2 ± 7.1	24.9 ± 10.2	0.621
Body temperature pre-ECMO, °C	36.8	37.1	0.598
Time from onset to ECMO initiation, h	9.0 (5.0–19.25)	3.0 (1.0–6.5)	0.002^*^
Duration of ECMO support, h	141.3 (72.0–288.0)	27.3 (8.8–87.5)	0.001^*^
Mode			0.008^*^
V-V ECMO	5 (27.8%)	1 (2.4%)	
V-A ECMO	13 (72.2%)	41 (97.6%)	
ECMO combined with CRRT	5 (27.7%)	6 (14.3%)	0.216
Outcome			0.389
Mortality	14 (77.8%)	28 (66.7%)	
Discharge	4 (22.2%)	14 (33.3%)	

*Data are mean ± standard, median (IQR), n (%). P-values were calculated by Mann-Whitney U-test, χ^2^ test, or Fisher's exact test, as appropriate. COPD, Chronic Obstructive Pulmonary Disease; CKD, Chronic Kidney Disease; APACHE, Acute Physiological and Chronic Health Evaluation; CRRT, Continuous Renal Replacement Therapy.*

* *P-value < 0.05*.

### Clinical Characteristics

We compared the main reasons for ECMO therapy between the two groups (Table 1). These included cardiogenic shock, respiratory failure, infectious shock, traumatic shock, electrical injury, intoxication, and aortic dissection, and there were no significant differences between the two groups. We also compared APACHE II score, pre-ECMO body temperature, time from onset to ECMO initiation, duration of ECMO support, ECMO mode, use or non-use of continuous renal replacement therapy (CRRT), and outcomes between the two groups. There were significant differences between the two groups in the time from onset to the ECMO initiation, duration of ECMO support, and ECMO mode. The duration of ECMO support and the time from onset to the ECMO initiation were higher in the hemorrhage group than in the no-hemorrhage group. The proportion of venoarterial (VA)-ECMO was significantly higher in the hemorrhage group than in the no-hemorrhage group.

### Hemorrhage-Related Organ Function Before the ECMO Initiation

We analyzed the organ damage in the two groups before the ECMO initiation, including cardiac arrest, hepatic injury, renal injury, and pulmonary infections (Table 2). The proportion of patients with the above organ injuries was similar between the hemorrhage and no-hemorrhage groups. However, pulmonary infections were noted in 7 (38.9%) and 4 (9.5%) cases in the hemorrhage and no-hemorrhage groups, respectively, before the ECMO initiation, and the difference between the two groups was statistically significant (*p* = 0.012).

**Table 2:** Hemorrhage-related organ function before the ECMO initiation.

**Variable**	**Hemorrhage Group (*n* = 18)**	**No-hemorrhage Group (*n* = 42)**	***P*** **-value**
Cardiac arrest	8 (44.4%)	19 (45.2%)	0.955
Pulmonary infection	7 (38.9%)	4 (9.5%)	0.012^*^
Liver injury	10 (55.6%)	28 (66.7%)	0.413
Renal injury	7 (38.9%)	8 (19.0%)	0.104

*Data are n (%).*

* *P-value < 0.05*.

### Laboratory Characteristics

Laboratory and point-of-care testing results on patients before the initiation of ECMO were collected, including routine blood, liver function, renal function, coagulation function, albumin, erythrocyte sedimentation rate (ESR), myocardial enzyme, and arterial blood gas tests (Table 3). There were significant differences between the two groups in red blood cell count, platelet count, ESR, alanine aminotransferase, and APTT (*p* < 0.05). The red blood cell count and platelet count were lower, and ESR, alanine aminotransferase, and APTT were higher in the hemorrhage group than in the no-hemorrhage group.

**Table 3:** Laboratory characteristics of patients.

**Variable**	**Hemorrhage Group (*n* = 18)**	**No-hemorrhage Group (*n* = 42)**	***P*** **-value**
White blood cell count, × 10^9^/L	15.5 (13.1–21.2)	12.0 (8.8–18.3)	0.084
Neutrophil count, × 10^9^/L	11.8 (10.9–15.1)	11.0 (7.4–14.1)	0.283
Lymphocyte count, × 10^9^/L	2.0 (1.4–3.8)	1.7 (0.8–3.1)	0.545
Red blood cell count, × 10^12^/L	4.19 (3.86–4.75)	4.43 (4.21–4.93)	0.019^*^
Hemoglobin, g/L	125.0 (113.5–137.0)	144.0 (135.8–161.8)	0.092
Platelet count, × 10^9^/L	138.4 ± 48.4	183.1 ± 68.5	0.003^*^
ESR, mm/h	34.5 (23.3–68.0)	8.0 (3.3–42.4)	0.013^*^
Alanine aminotransferase, IU/L	118.0 (46.5–256.3)	36.5 (14.5–201.5)	0.045^*^
Aspartate aminotransferase, IU/L	181.5 (62.0–388.8)	114.0 (51.8–565.5)	0.821
Serum creatinine, μ mol/L	198.7 (145.9–239.4)	111.6 (89.4–158.3)	0.191
Lactate dehydrogenase, IU/L	675.5 (279.8–1,380.5)	595 (404.8–1,340.5)	0.463
Creatine kinase, IU/L	1,201.5 (329.5–1,659.3)	998.0 (356–2,325.5)	0.634
α-HBDH	357.0 (222.5–900.8)	332.5 (214.8–655.0)	0.711
Albumin, g/L	28.5 (24.0–31.2)	32.1 (25.5–36.8)	0.223
APTT, s	71.3 (44.1–105.9)	39.9 (29.7–58.8)	0.015^*^
PT, s	25.9 (18.7–29.2)	12.1 (9.3–15.5)	0.578
D-Dimer, μ g/ml	4.0 (0.7–10.5)	1.8 (0.8–14.7)	0.503
Fibrinogen, g/L	3.1 (2.6–4.7)	2.9 (2.2–3.6)	0.242
PH	7.208 (7.106–7.242)	7.226 (7.129–7.257)	0.519
PaO_2_, mmHg	68.6 (45.0–72.5)	73.2 (64.0–82.0)	0.103
PaCO_2_, mmHg	63.6 (47.5–69.8)	59.5 (47.5–84.6)	0.217
Lactate, mmol/L	8.73 (4.88–11.10)	7.70 (5.0–9.6)	0.384

*Data are mean ± standard, median (IQR). P-values were calculated by Mann-Whitney U-test, χ^2^ test, or Fisher's exact test, as appropriate. ESR, Erythrocyte Sedimentation Rate; α-HBDH, Alpha-hydroxybutyric Dehydrogenase; APTT, Activated Partial Thromboplastin Time; PT, Prothrombin Time.*

* *P-value < 0.05*.

### Logistic Regression

**Figure 1:**
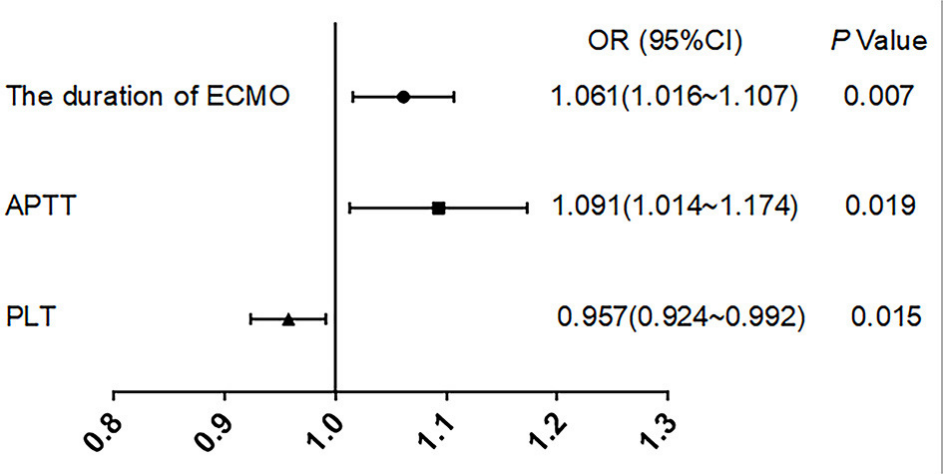
Forest plot.

We included 60 patients with complete data for all variables in the binary logistic regression model *via* stepwise regression (Table 4). We found that duration of ECMO support (OR = 1.061, 95% CI: 1.016–1.107, *p* = 0.007), APTT (OR = 1.091, 95% CI: 1.014–1.174, *p* = 0.019), and platelet count (OR = 0.957, 95% CI: 0.924–0.992, *p* = 0.015) were independently associated with hemorrhagic complications in adults on ECMO. The logistic regression results were shown in the forest plot (Figure 1).

**Table 4:** Logistic regression analysis for the related factors predicting hemorrhage.

**Variable**	**B**	**S.E**.	**Wals**	**OR**	**95%CI**	***P*** **-value**
Duration of ECMO support, h	0.059	0.022	7.258	1.061	1.016–1.107	0.007
APTT, s	0.087	0.037	5.461	1.091	1.014–1.174	0.019
Platelet count, × 10^9^/L	−0.044	0.018	5.928	0.957	0.924–0.992	0.015

*B, partial regression coefficients; S.E., standard error; OR, odds ratios; CI, confidence interval*.

### ROC Curves

**Figure 2:**
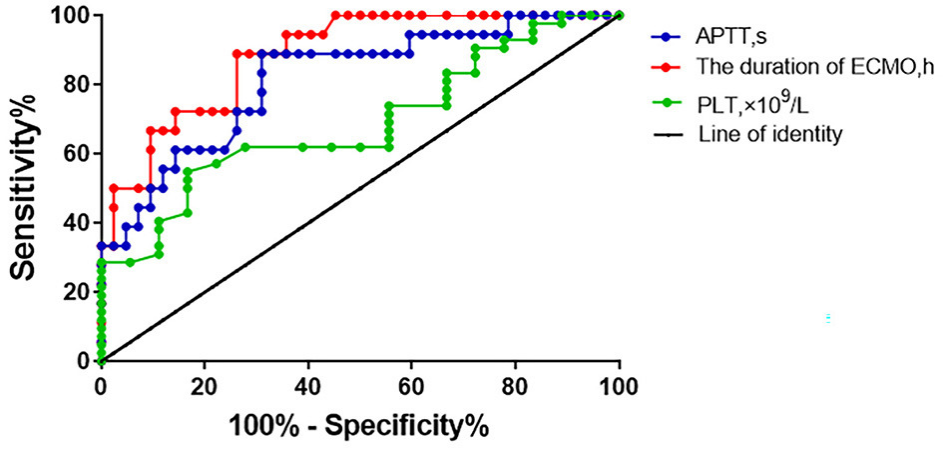
ROC curves of logistic model.

The ROC curves were used to evaluate the predictive value of the binary logistic model (Figure 2). The area under the ROC curve (AUC) for the duration of ECMO support was 0.884 (*p* < 0.001). The sensitivity and specificity of ECMO duration were 88.9 and 73.8%, respectively, and the cutoff value for ECMO duration was 62.70 h. For APTT, the AUC was 0.817 (*p* < 0.001), the sensitivity and specificity were 83.3 and 69.0%, respectively, and the cutoff value was 52.3 s. Regarding platelet count, the AUC was 0.751 (*p* = 0.002), the sensitivity and specificity were 83.3 and 54.8%, respectively, and the cutoff value was 38.10 × 10^9^/L (Tables 5, 6).

**Table 5:** ROC curve analysis and characteristic parameters.

**Variable**	**AUC**	**S.E**.	***P*** **-value**	**95%CI**
Duration of ECMO support, h	0.884	0.043	0.000	0.800~0.968
APTT, s	0.817	0.060	0.000	0.699~0.935
Platelet count, ×10^9^/L	0.751	0.063	0.002	0.627~0.874

*AUC, The area under the ROC curve*.

**Table 6:** Cutoff value, sensitivity and specificity of ROC curve.

**Variable**	**Cutoff value**	**Sensitivity (%)**	**Specificity (%)**
Duration of ECMO support, h	62.70	88.9	73.8
APTT, s	52.30	83.3	69.0
Platelet count, ×10^9^/L	38.10	54.8	83.3

### Analysis of Hemorrhage-Related Indicators in the Hemorrhage Group

**Figure 3:**
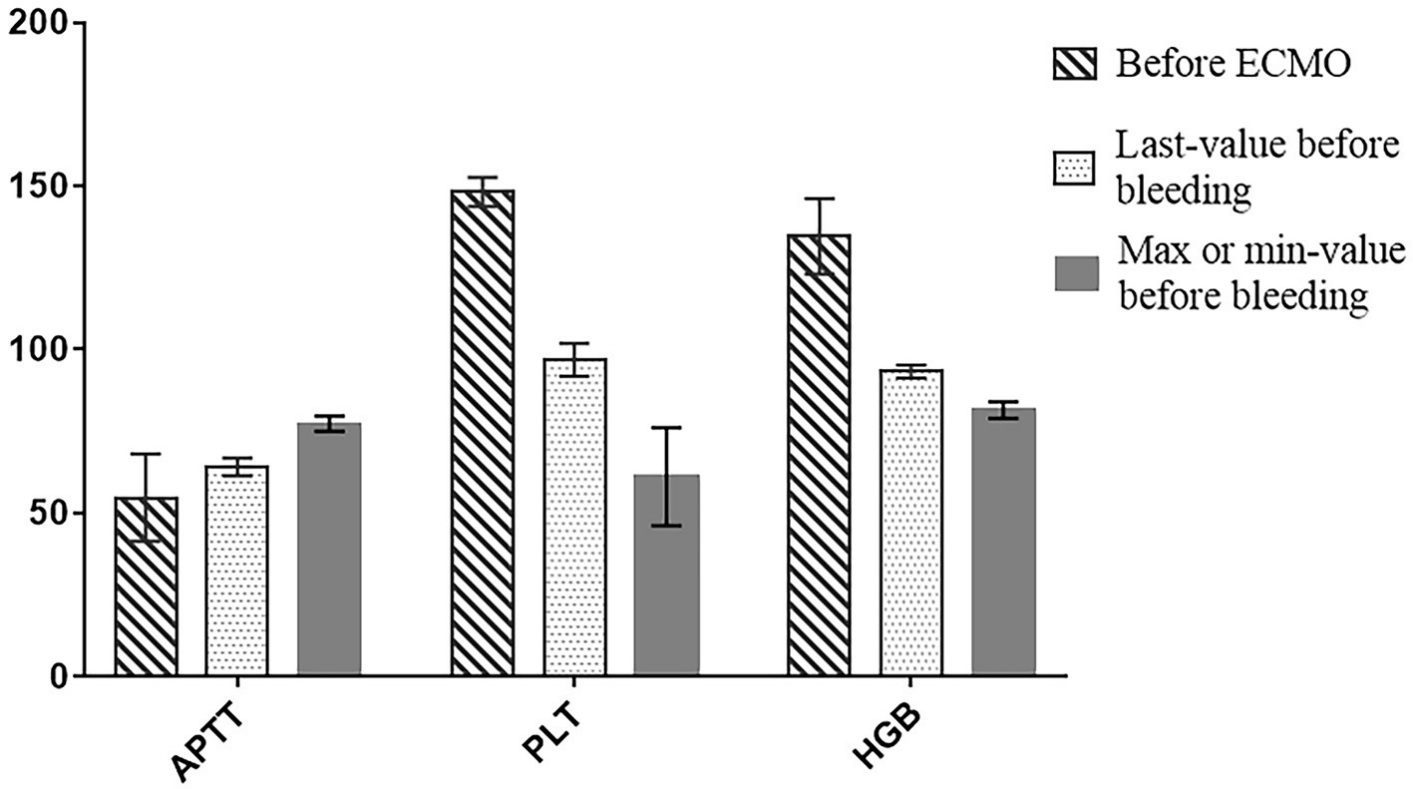
Column diagram of APTT, PLT and HGB.

Each patient was tested for coagulation function once every 2–6 h, and routine blood examination was repeated at least once a day. We recorded the maximum APTT, minimum platelet count, and minimum hemoglobin values before the bleeding event and the mean values of APTT, platelet count, and hemoglobin at the last time before bleeding in the hemorrhage group (Table 7). We compared the mean values of APTT, platelet count, and hemoglobin at these two time points with the mean values of APTT, platelet count, and hemoglobin before going on the ECMO machine (Figure 3). After the ECMO initiation, the patients showed a tendency to have a prolonged APTT and decreased platelet count and hemoglobin.

**Table 7:** Analysis of hemorrhage-related indicators in the hemorrhage group.

**Variable**	***n*** **= 18**
Max-APTT, s	77.33 ± 17.28
Min- Platelet count, ×10^9^/L	61.22 ± 17.38
Min- Hemoglobin, g/L	81.50 ± 11.36
Last-APTT, s	64.16 ± 25.07
Last- Platelet count, ×10^9^/L	96.83 ± 18.42
Last- Hemoglobin, g/L	93.28 ± 9.77

*Data are mean ± standard*.

### Blood Transfusions

Blood transfusion during this treatment was recorded in both groups (Table 8). The amount of blood transfusion was significantly higher in the hemorrhage group than in the no-hemorrhage group. The amount of transfused plasma was 2,075.0 (900.0–4,281.25) ml in the hemorrhage group, which was significantly higher than the 1,100.0 (400–2,350.0) ml in the no-hemorrhage group (*p* = 0.041). The amount of transfused PRBC was 8.75 (2.0–23.50) units in the hemorrhage group, which was significantly higher than the 4.0 (2.0–7.75) units in the no-hemorrhage group (*p* = 0.031). However, there was no significant difference between the two groups regarding platelet transfusion (*p* > 0.05).

**Table 8:** Blood transfusions in the whole course of treatment.

**Blood products**	**Hemorrhage group (*n* = 18)**	**No-hemorrhage group (*n* = 42)**	***P*** **-value**
Plasma, ml	2,075.0 (900.0~4,281.25)	1,100.0 (400~2,350.0)	0.034^*^
PRBC, U	8.75 (2.0~23.50)	4.0 (2.0~7.75)	0.031^*^
Platelet, therapeutic dose	2.0 (0.75~4.0)	0 (0~2.0)	0.07

*Data are median (IQR).*

* *P-value < 0.05*.

### Hemorrhagic Sites and Time From ECMO Initiation to Hemorrhage

**Figure 4:**
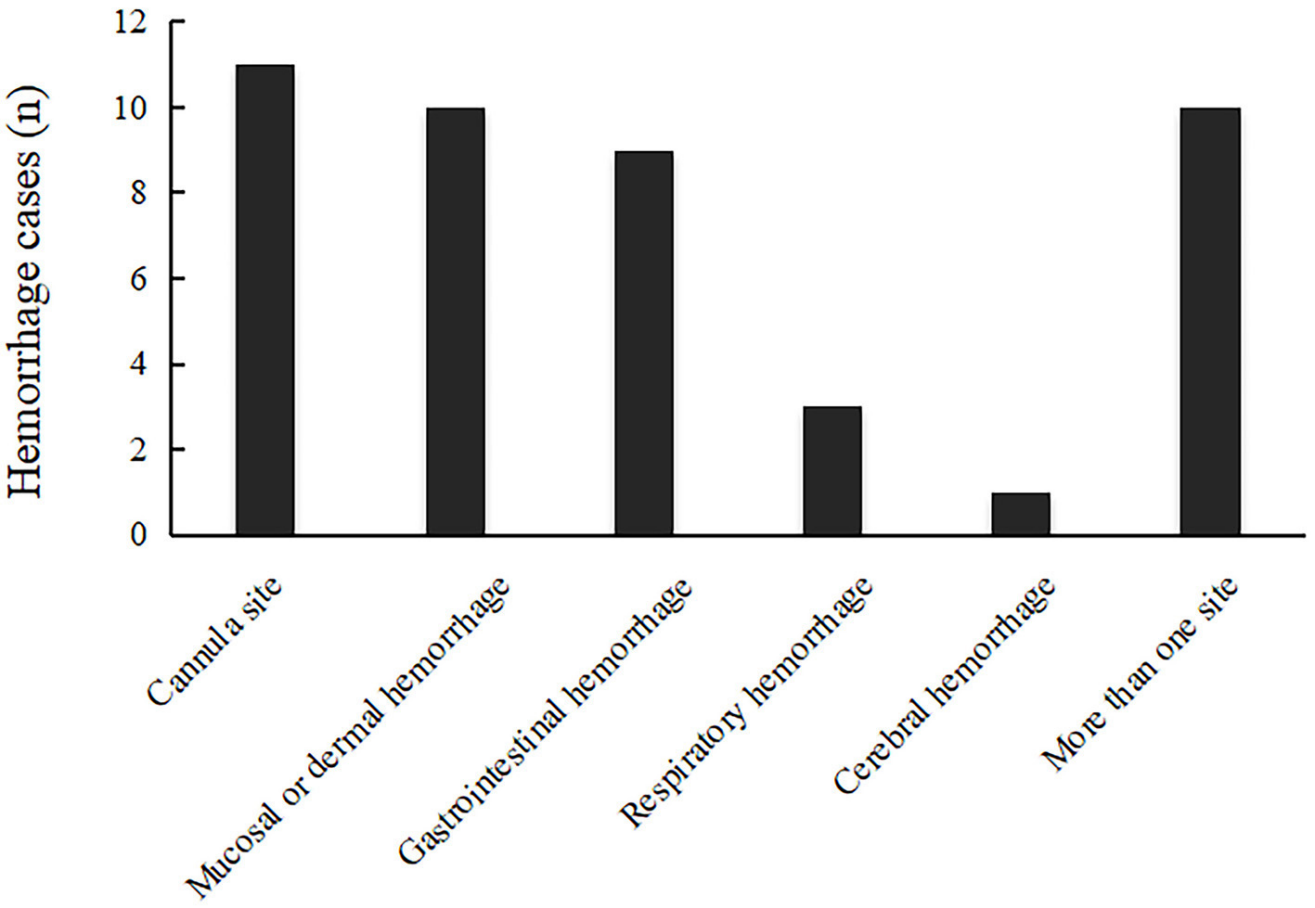
Hemorrhage sites.

About 10 patients (55.6%) in the hemorrhage group had more than one hemorrhagic site (Table 9; Figure 4). The most prevalent hemorrhagic complication was cannula site bleeding [*n* = 11 (61.1%)]. Mucosal or dermal hemorrhage [*n* = 10 (55.6%)] and gastrointestinal hemorrhage [*n* = 9 (50%)] were also common. The mean time from ECMO initiation to hemorrhage was 2.1 ± 0.96 days.

**Table 9:** Hemorrhage sites and time from ECMO initiation to hemorrhage.

**Variable**	**Total (*n* = 18)**
**Hemorrhage sites**	
Cannula site	11 (61.1%)
Mucosal or dermal hemorrhage	10 (55.6%)
Gastrointestinal hemorrhage	9 (50.0%)
Respiratory hemorrhage	3 (16.7%)
Cerebral hemorrhage	1 (5.6%)
More than one site	10 (55.6%)
Time from ECMO operated to hemorrhage, days	2.1 ± 0.96

*Data are mean ± standard and n (%)*.

## Discussion

Extracorporeal membrane oxygenation is a proven life-saving treatment for patients with respiratory and cardiac failure. However, mortality is still high during ECMO. Hemorrhagic complications are frequent and are an important factor, limiting both therapeutic efficiency and patient survival. In studies of adult patients on ECMO, the incidence of hemorrhagic complications varied between 15 and 63% (6–8). In these studies, regarding anticoagulation in venovenous (VV)-ECMO (*n* = 646), the overall rate of hemorrhagic complication was 29%, whereas major bleeding rates across the studies were 10–16% (9, 10). The necessity of anticoagulants and complicated pathophysiological changes of the coagulation cascade in critically ill patients present a substantial challenge in reducing the incidence of bleeding events. Clinicians should consider potential risk factors of hemorrhage prior to ECMO initiation.

In our study, 72.2% of the patients in the hemorrhage group used VA-ECMO, and 27.8% used VV-ECMO. Half of the patients in the bleeding group had a history of hypertension, nearly half had a history of cardiac disease (coronary artery disease, valvular disease, atrial fibrillation, etc.), and one-third had a previous history of anticoagulation. The main diagnosis for patients with bleeding was cardiogenic shock, followed by infectious shock and respiratory failure. More than half of the patients had varying degrees of cardiac, hepatic, renal, pulmonary, and other-organ damage before ECMO, including 55.6% with liver damage and 44.4% who had experienced cardiac arrest. Most patients in the hemorrhage group had reductions in hemoglobin concentration, erythrocyte count, and platelet count, and impaired liver and kidney function compared to the no-hemorrhage group. APTT tended to be prolonged, and platelet count and hemoglobin tended to be decreased in the hemorrhage group after ECMO was initiated. The hemorrhage group also had more requirements for transfusion during the treatment period than the no-hemorrhage group.

Bleeding sites may include cannula insertion sites, recent surgical incisions, dermal or mucosal region, lung, gastrointestinal tract, and brain (11). The ELSO guidelines reported that the cannulation site is the most common site of bleeding, particularly if access is gained by direct cutdown (12). This finding was also noted in our study. All patients in our study *via* post-operative cannulation. The most prevalent hemorrhagic complication in our study was cannula site bleeding (61.1%). The rate of mucosal and dermal hemorrhage was 55.6%, and the rate of gastrointestinal hemorrhage was 50.0%. Blood vessel and skin injury caused by the cannula cannot be avoided. Usually, cannula site bleeding is slow-oozing and is related to the disruption of small vessels in the skin or subcutaneous tissue. Topical pressure often controls the bleeding in these areas. When the body is in the stress state of ischemia and hypoxia, the blood redistributes. Then, stress ulcer occurs in the gastrointestinal tract due to insufficient blood supply, decreased mucosal blood flow, ischemia, and reperfusion injury. The stress ulceration can progress and erode larger vessels resulting in overt gastrointestinal bleeding (13). This may be the main cause of gastrointestinal bleeding in patients on ECMO. Intracranial hemorrhage (ICH) and respiratory hemorrhage were rare in our study, but both are serious complications. However, as patients who received ECMO are commonly in a moderate-to-deep coma, it may be challenging to diagnose ICH early (14). Clinical manifestations of ICH notably vary, depending on the cerebral structures affected. ICH may present with focal sensorimotor deficits, seizures, pupillary abnormalities, coma, or brain death. It is important to perform a physical examination and computed tomography. Small traces of upper respiratory hemorrhage may be due to endotracheal intubation and tracheal suction, and a properly performed procedure may prevent this occurrence. Pulmonary alveolar hemorrhage may cause acute respiratory distress syndrome in patients which is a major clinical problem (15). Severe bleeding events may lead to the abrupt cessation of anticoagulation and increase thromboembolic risk. Thus, balancing the risk of thrombosis and bleeding in the management of such patients is important but challenging. The mean time from the ECMO initiation to hemorrhage was 2.1 days, showing that preventing bleeding events within the first 3 days is crucial.

We confirmed three risk factors for hemorrhage in adults on ECMO. In particular, prolonged ECMO support, a high pre-ECMO APTT, and a low pre-ECMO platelet count were independently associated with a higher incidence of hemorrhage. The cutoff value of APTT was 52.3 s, which indicates that patients with APTT > 52.3 s before ECMO is initiated have a significantly high risk of bleeding. APTT is the time (in seconds) in which calcium-free plasma clots in response to a fibrin-activating reagent combined with calcium. APTT is primarily used to monitor and titrate heparin dosing. The ELSO guidelines (2014) recommended that patients with a low bleeding risk should be maintained at ACT 180–200 s or APTT 60–80 s (or 1.5 times the basal value). Patients with a high bleeding risk should be maintained at a flow of >3 L/min, ACT 160 s, or APTT 45–60 s. Patients with active bleeding should be maintained at a flow of >3 L/min, with cessation of heparin anticoagulation and close monitoring of ACT, APTT, membranopulmonary and ductal thrombosis, and thrombogenesis. Thromboelastography should be performed daily to assess the risk of coagulation (3). Our findings are in line with a retrospective study of 164 patients who received ECMO support, in which the highest APTT quartile on the day prior to the bleeding event was shown to be an independent risk factor (4). Administration of heparin, as reflected by the level of anticoagulation (APTT target), is modifiable. High APTT was associated with the occurrence of hemorrhagic complications. A low APTT target range may reduce bleeding events, but at the same time, may also increase thrombotic events (16). Hence, an appropriate APTT target range is the key to balancing anticoagulation and antithrombosis. Some studies found that there is no association with measured parameters of anticoagulation and observed bleeding and thrombosis. However, more evidence shows that ACT, APTT, and thromboelastograms are valuable and recommended by the ECMO guidelines.

The cutoff value for the duration of ECMO support was 62.70 h, indicating that an ECMO duration exceeding 62.70 h may be associated with a significantly high risk of bleeding. A study showed that the ECMO duration in patients with ICH was approximately twice that of those without ICH and was an independent risk factor for ICH occurring during ECMO support (17). This is probably related to the longer exposure to anticoagulant medications, which are crucial for all patients on ECMO. Similarly, the current study showed that the duration of ECMO support was an independent risk factor for hemorrhage in adults on ECMO. A longer ECMO duration means longer exposure to anticoagulant medications. Therefore, there is a higher chance of exposure to supratherapeutic levels of anticoagulants. However, systemic anticoagulation, the extracorporeal circuit, and complicated illnesses make it difficult to reduce the duration of ECMO. We recommend more frequent monitoring of coagulation indicators (especially ACT and APTT) in patients who remain on ECMO for prolonged periods and to attempt earlier rather than later weaning from ECMO whenever feasible to avoid the risk of bleeding.

A decreased platelet count has been shown by several studies to be a risk factor for bleeding in ECMO patients (8, 18, 19). Platelets play an important role in physiological hemostasis. There are many reasons for the reduced platelet count in ECMO patients, including damage to platelet function from blood flow shear in the tubing, depletion of platelets by non-biological materials, multiple causes of vascular hemophilia factor reduction, and heparin-induced thrombocytopenia (18). Our study suggests that the risk of bleeding in patients decreased as platelet count increased. The cutoff value for platelet count was 38.1 × 10^9^/L. Therefore, the platelet counts below 38.1 × 10^9^/L before ECMO seem associated with a significantly high risk of bleeding. A study found that platelet count was notably reduced in the first 3 days after the ECMO initiation, with a more gradual but still progressive decrease from days 3 to 7 (18). It is important for clinicians to detect a reduction in platelet count as early as possible in order to promptly intervene and replenish the platelet count. “The Expert Consensus on the Diagnosis and Treatment of Thrombocytopenia in Adult Critically Ill Patients in China (2020 version)” published by the Chinese Medical Association recommends that platelet transfusion be started in patients for ECLS with platelet count <50 × 10^9^/L (19). However, the threshold for platelet transfusion in our hospital is 30.0 × 10^9^/L.

In our study, the platelet count decreased significantly after the ECMO initiation, and the difference in platelet count between the hemorrhage and non-hemorrhage groups was statistically significant. However, the difference in the amount of platelet transfusion between the two groups was not statistically significant. We believe that increasing the threshold value for platelet transfusion may reduce bleeding events in adults on ECMO.

## Limitations

Our study has several limitations. First, it was a single-center retrospective study. Our hospital did not have enough patients on ECMO to conduct a large-scale clinical study. Thus, we could only explore the association between certain risk factors and hemorrhagic complications. Second, we do not routinely use thromboelastography, platelet function, and anti-Xa factor analysis in our hospital, which are important anticoagulation indicators and might have provided additional information. A larger prospective study separately considering fatal bleeding as an outcome should also be meaningful.

## Conclusion

Patients receiving ECMO support who have hemorrhagic complications are subject to risk from their coagulation function, underlying disease, and duration of ECMO support. This study showed that patients with platelet count <38.1 × 10^9^/L, APTT longer than 52.3 s before ECMO, and duration of ECMO treatment longer than 62.7 h have a significantly high risk of bleeding. Cannula sites, the mucosa, skin, and gastrointestinal tract are the common sites of bleeding. There should be a greater focus on these sites to prevent hemorrhagic complications. The frequent monitoring of coagulation indicators in the first 3 days of ECMO and timely weaning from ECMO support are sensible and crucial. In addition, increasing the threshold value for platelet transfusion may reduce bleeding events in adults on ECMO.

## Data Availability Statement

The original contributions presented in the study are included in the article/supplementary material, further inquiries can be directed to the corresponding author/s.

## Ethics Statement

The studies involving human participants were reviewed and approved by Human Ethics Committee and the Research Ethics Committee of Taihe Hospital, Hubei, China. Written informed consent for participation was not required for this study in accordance with the national legislation and the institutional requirements. Written informed consent was obtained from the individual(s) for the publication of any potentially identifiable images or data included in this article.

## Author Contributions

WH contributed to data curation, formal analysis, and writing of original draft preparation. JZ and MW performed methodology and project administration. WC involved in investigation and data curation. LC performed formal analysis and software. EL helped in writing, reviewing, and editing. YT contributed to the supervision, project administration, and funding acquisition. All authors contributed to the article and approved the submitted version.

## Funding

This work was supported by Hubei Province's Outstanding Medical Academic Leader Program and the Innovation Team of Hubei University of Medicine (FDFR201801).

## Conflict of Interest

The authors declare that the research was conducted in the absence of any commercial or financial relationships that could be construed as a potential conflict of interest.

## Publisher's Note

All claims expressed in this article are solely those of the authors and do not necessarily represent those of their affiliated organizations, or those of the publisher, the editors and the reviewers. Any product that may be evaluated in this article, or claim that may be made by its manufacturer, is not guaranteed or endorsed by the publisher.

## Abbreviations

ACT: activated clotting time
APACHE: Acute Physiology and Chronic Health Evaluation
APTT: activated partial thromboplastin time
CRRT: continuous renal replacement therapy
ECLS: extracorporeal life support
ECMO: extracorporeal membrane oxygenation
ELSO: Extracorporeal Life Support Organization
ESR: erythrocyte sedimentation rate
ICH: intracranial hemorrhage
PRBC: packed red blood cell
UNFH: unfractionated heparin
VA: venoarterial
VV: venovenous.
